# “They make money off of us”: a phenomenological analysis of consumer perceptions of corruption in Kenya’s HIV response system

**DOI:** 10.1186/s12913-016-1721-y

**Published:** 2016-09-05

**Authors:** Njeri Kagotho, Alicia Bunger, Kristen Wagner

**Affiliations:** 1 Ohio State University College of Social Work, 325-E Stillman Hall, 1947 College Road, Columbus, OH 43210 USA; 2 University of Missouri St. Louis, 121 Bellerive Hall, One University Boulevard, St. Louis, MO 63121 USA

**Keywords:** HIV/AIDS, Corruption, Kenya, Phenomenological study

## Abstract

**Background:**

Problems with misallocation and redirection of critical resources and benefits intended for PLHIV are not uncommon in Kenya. This study explores corruption in Kenya’s HIV response system and the implications for health outcomes from the perspective of people living with HIV (PLHIV). Although they might not be directly responsible for health care fund management, PLHIV and their advocacy efforts have been central to the development of HIV system response and they have a vested interest in ensuring proper governance.

**Methods:**

This phenomenological study was conducted in 2012 in Kiambu County in Kenya. The study was designed to capture the experiences of a select group of individuals living with HIV and AIDS and subsequent effects on intergenerational wealth transmission. Four focus groups were conducted with self-convened HIV/AIDS peer support groups. Findings related to corruption emerged unexpectedly, albeit consistently, across all four focus groups. To validate core themes within the data, including corruption, two coders independently reviewed and coded the data.

**Results:**

Participants described incidences of resource misallocation, theft, and denial of services across three thematic levels namely at the interpersonal, provider, and institutional levels. Participants described the negative influence of corruption on their health and financial well-being, and propose: (1) strengthening legal protections for assets belonging to PLHIV, (2) direct representation of PLHIV within service agencies, (3) and addressing information asymmetries to inject transparency into the response system.

**Conclusion:**

Our findings add to the growing literature that identifies advocacy among individuals and families impacted by HIV and AIDS to be a useful tool in drawing attention to harmful practices in the HIV response infrastructure; consistent with this movement, communities in Kenya demand greater control over programmatic interventions both at the national and local levels.

## Background

Kenya continues to have one of the highest HIV prevalence rates in the world [1]. High prevalence and HIV/AIDS related deaths prompted the Kenyan government to declare HIV a national disaster in 1999. Although the rate of new HIV/AIDS infections in Kenya has declined steadily since the late 1990s, when the highest rates were recorded [2, 3], 1.6 million Kenyans are reportedly living with HIV [3]. Kenya’s HIV response system is multi-sectorial in nature, characterized by a network of services provided through an array of Ministries and State Departments, County Governments, and various arms of the private and corporate sectors [3]. This broad and overarching response system uses a social justice perspective to integrate prevention and health services, research and development, and organizational development [3]. At the national level, financing and administration of testing and treatment services is the responsibility of the Ministry of Health through The National AIDS and STI Control Program (NASCOP). Other government ministries—such as the Ministry of Labour, Social Security, & Services; Ministry of Education, Science, & Technology—are responsible for administering and delivering auxiliary services such as economic empowerment supports for vulnerable families, education bursaries for children orphaned or made vulnerable by AIDS, and enforcement of anti-discrimination policies.

Although the Kenyan government has developed public agency infrastructure for delivering HIV services, only 18 % of these expenditures are financed through public coffers. Rather, the extensive healthcare needs have meant that the lion’s share of the HIV response costs are borne by international development partners, the corporate sector, and private households [4]. It was estimated that Kenyans spent over US$ 98 million on out of pocket costs in HIV related care in 2011/12 accounting for 13 % of the response system. PLHIV spend approximately seven times more than what the average Kenyan spends on health care [4] which includes medical costs associated with HIV and HIV related illnesses. In 2012, the private and corporate sectors contributed approximately US$ 15.93 million to the HIV response system. They do this providing work place programs such as interventions dealing with stigma and gender relations, pharmaceutical treatment, and inpatient and outpatient care [4]. Finally, international development partners play an integral part in the HIV response system by bearing a significant share of the cost burden—approximately 70 % between 2009/12. This over – reliance on international donor supports raises concerns about the Kenya government’s ability to sustain the response effort.

### Corruption in Kenya

Problems with misallocation and redirection of critical resources and benefits intended for PLHIV are not uncommon in Kenya, where corruption is widespread. In a worldwide corruption perceptions index, Kenya was ranked 139 out of 168 nations, with 43 % of those surveyed perceiving corruption as a problem in the health care sector alone [5]. Corruption is defined as public servants’ abuse of their position for personal gain [6]. Kenyans have extensive personal experience with corruption; 77 % of those surveyed about corruption in Kenya reported that someone in their households paid a bribe to the police, 54 % to public registry departments, and 35 % to medical healthcare providers [5]. Within health care and other public systems that support PLHIV, corruption can also manifest as a variety of behaviors among decision makers within public agencies, provider facilities, and community groups. Other examples include kickbacks for procurement decisions, embezzlement, fraudulent claims of services rendered, theft of funding or supplies, charging individuals for services or products that should be free, providing poor quality care, diverting individuals away from public benefits, and excessive absenteeism among providers [7–9]. Bribes and corrupt behaviors may be used both by consumers as a conduit to access legitimate services or by decision makers to gain access to unmerited goods and services.

Regardless of the level at which it occurs, corruption obstructs the delivery of resources, services, medications, and benefits to those who are most in need, unduly impacting low-income Kenyans, and can lead directly to poor health outcomes [10, 11]. Unchecked corruption threatens the stability of international aid, health programming [12], and national economic growth [13]. In addition, corruption can lead to distrust in public institutions and healthcare providers. Especially in healthcare, where patients rely on professionals to consider their best interests, distrust limits individuals’ motivation and likelihood of accessing needed services, and compromises personal health [14, 15].

### Conditions that drive corruption

Although corruption among public officials is often driven by individual factors, their behavior is also heavily influenced by organizational, institutional, and cultural contexts [6, 16]. In many cases, corruption is motivated by the potential for personal financial gains, friendship, or family, but made possible by ample opportunity to engage in these behaviors without being held accountable [6]. As large sums of international aid are dispersed throughout complex public systems, weak governance infrastructure, limited integrity policies, poor supervision, and wide ranging personal discretion in decision making allow public officials to misdirect resources without being detected [7, 16]. Another factor may be that decision makers feel justified in accepting a bribe because they feel their work is inadequately rewarded [17].

Furthermore, personal, organizational, or cultural values that enable public officials to rationalize their behavior can drive corruption. For instance, weak ethical values, strong social norms, or cultural taboos such as the social stigma associated with HIV/AIDS could legitimate corrupt behaviors [6]. Kenya’s political structure and ethnic heterogeneity has been viewed as a driver of corruption. Kenya has had a long history of political patronage driven by ethnic and regional motivations. The result has been economic inequalities along ethnic, and by extension, geo-political boundaries [18, 19]. These inequalities have bred an environment of ethnic cronyism where individuals engage in corrupt activities not purely for private gain but also for the benefit of fellow tribesmen. Indeed, Orjuela [20] cautions that in societies where group identity is paramount, corrupt behaviors and actions in pursuit of “personal again” could extend to include the “collective gain” of a particular group.

Fighting corruption often requires multiple complementary measures that target internal governance, improve transparency, and engage the public in promoting accountability. Internal accountability mechanisms that tighten personnel management, procurement, and oversight processes can reduce the opportunity to misallocate resources. For instance, instituting staff performance appraisals, codes of ethical conduct, mechanisms for reporting and subsequent sanctions for misconduct, and financial incentives for performance could minimize opportunities, pressures, and acceptance of corruption [9]. To complement internal governance initiatives, external mechanisms are needed that engage the public in promoting accountability among systems, providers, and other decision makers. Provider report cards, consumer advocacy or advisory groups, complaint boxes, and health facility charters that advertise the cost of services can promote transparency and information dissemination to the community, empowering patients to challenge corruption [21–23].

### Consumer advocacy and the global HIV response

In 1978, the World Health Organization introduced the idea of local involvement and control in the design and implementation of health systems as a necessary mechanism to effectively address public health issues, particularly in the global South [24]. UNAIDS guidelines requiring community engagement were established in 2000. Initially, engagement was focused on research participation and protections through Community Advisory Boards (CAB) but engagement has since expanded to more broad participation in research, advocacy, program and policy design, and service delivery. Effective community engagement processes are based on: 1) authentic participation of a representative group of stakeholders, 2) mutual trust between community and partners, and 3) shared power [25]. Engaged community may lead to greater transparency in the research and implementation processes [26], re-direction of resources to areas of highest need, [27, 28] and locally appropriate services [29]. Consumer advocacy is also associated with increased patient trust in providers. As consumers develop stronger trust in healthcare professionals, they may be more likely to comply with treatment [26, 30]. From a service provision perspective the use of unpaid volunteers in service provision is geared towards increasing the chances of program sustainability. It should be noted that community volunteers account for approximately 40 % of community based organizations’ (CBO) budgets. This is especially salient given that in Kenya funding most likely comes from external donors and is limited (average annual budgets equal approximately US$ 15,000) [31]. However, reliance on under- or uncompensated labor, particularly in communities where needs are great, could be construed as exploitative and is indeed unsustainable in the long run [32]. Thus, even in systems with strong consumer advocacy and involvement, there still remains a strong risk of the misalignment of resources to local needs.

## Methods

This study explores consumers’ experiences in Kenya’s HIV response system and describes corruption and implications for personal health and well-being. Consumers [1] describe instances of corrupt behavior at multiple levels of the system, [2] explain the effect of corruptive behaviors on personal health and well-being, and [3] identify potential strategies for improving governance and accountability within the healthcare system.

This phenomenological study was conducted in 2012 in Kiambu, Kenya. Phenomenology allows for the exploration of lived experiences. Unlike grounded theory which focuses on the objective generation or discovery of theory, the phenomenological approach seeks to unearth the deeper meaning of experiences solely from the perspective of an individual’s life experiences [33, 34]. Furthermore, this technique aims to reduce the degree to which conceptualizations of the phenomena are superimposed on research participants’ re-telling and understanding of their experiences. Given the dearth of information on the phenomenon of intergenerational wealth transmission in households at the confluence of poverty and chronic illness, this study was originally designed to capture these experiences among a select group of heads of households. Four focus groups were conducted with self-convened HIV/AIDS peer support groups in the region. In light of the original study’s focus on personal and family asset holdings, the findings related to corruption presented in this manuscript emerged unexpectedly, although consistently, across all four focus groups.

### Study design, setting, and population

Kiambu County is home to a mixed economy, with industries and large scale farms on the one hand and small subsistence land holdings and informal housing settlements (slums) on the other. The County Integrated Poverty Plan 2013-17 places the county’s poverty rate at 21.75 %. Kiambu county’s HIV prevalence rate stands at 3.8 % and ranks 38^th^ among Kenya’s 47 counties [35]. Admittedly, Kiambu County has inadequate health care services with a doctor/patient ratio of 1:17000 and the nurse/patient ratio at 1:1300. In addition the county has one level-five hospital, namely, Thika District Hospital [36]. Although located in a region predominated by the Kikuyu tribe, the county’s proximity to the capital city Nairobi and its peri-urban economy means that it is home to Kenyans from all ethnicities. Kenya has at least 40 different ethnic tribes each with unique intergenerational wealth transmission norms and rules. We documented understanding of this issue from different cultural perspectives by deliberately seeking peer support groups that were as culturally diverse as possible, although homogeneity was not an exclusion criteria.

A community gatekeeper assisted with recruitment by publicizing the study among local support groups. The gatekeeper had access to these groups through her participation with the local Constituency AIDS Control Committee (CACC). CACC’s are local bodies charged with the coordination of HIV and AIDS activities. HIV Peer support groups are required to register with their local CACC [37]. Participating groups were those that were able to convene with at least ten group members present and had to be able to commit at least two hours to the interview.

These recruitment procedures yielded a diverse sample, with regard to age, gender, and tribe. Of the 45 respondents 67 % were female. The respondents ranged in age from 28 to 63 years (M = 42.9, SD = 8.52). Household size ranged from 2 to 9 individuals (M = 4.9, SD =2.02) and with the exception of four respondents, all others were living in a household with at least one child under the age of 18 years. All respondents self-identified as major decision makers in their households. Only eight (18.6%) of this sample had completed secondary school, while nine (20.9 %) had not completed primary school education. Participants self-identified as Kamba, Kikuyu, Kisii, Luhya, and Luo thereby providing a diverse cultural perspective.

### Data collection and analysis

Data were collected through four in-depth focus group interviews and a brief six question close-ended survey with each respondent participating in only one focus group discussion. Focus group interviews are especially advantageous as a research method as a heterogeneous group is more likely to elicit lively conversations that lend to comparing and contrasting experiences, thereby providing a nuanced description of the phenomenon.

Interviews were conducted in Kiswahili (national language), English, Kikuyu, and Sheng (slang). The first author is fluent in all of these languages. Focus group interviews were digitally recorded with each groups’ permission, and flip charts used to highlight the key words and major points that emerged throughout each interview session. A research assistant (with certification in human subjects’ research) was also present to assist with facilitation. Transcriptionists were hired to transcribe and translate the data into English. The first author checked all transcriptions and translations to ensure accuracy and consistency.

Data were analyzed using AtlasTi software [38]. A phenomenological data analysis approach was used [33, 34]. To enhance the process, two coders—the first author (coder #1) and the second author (coder #2) independently reviewed the transcripts. It should be noted that coder #1 is Kenyan, has previously analyzed these data for other themes, and has been immersed in this literature for close to a decade while coder #2 is North American and has substantial experience working with qualitative data. To check for and avoid the introduction of assumptions, interpretations, and meanings exogenous to the transcripts as provided by the participants, both coders independently engaged in a neutral appraisal of the transcripts to establish a global understanding of the interviews. This process ensured completeness because each coder would ideally identify meaning units on account of their different theoretical backgrounds, world view and biases. In the second round of analysis, both coders met to discuss the emergent meaning units and to integrate these units into meaningful structures. After several iterations, this part of the data analysis process identified two levels at which corruption of resources takes place, namely provider and institutional levels. The final round of data analysis deconstructed the data further by attempting to understand what these different levels of misappropriation tell us about this population’s experiences. Connecting these two themes to the numerous examples in the data, the coders were able to explain this phenomenon by exemplifying how misappropriation takes place, the consequences on well-being, and suggested solutions to address the identified gaps.

## Results

The issue of corruption as it relates to the well-being of the groups was mentioned and discussed throughout all four focus groups. Participants described incidences of resource misallocation, theft, and denial of services across two thematic levels namely at the provider and institutional levels. Under each thematic level, quotations derived from respondents’ perceptions of how corruption manifests in their lives, their interpretation of its impact on overall well-being, and proposed solutions are presented. Table 1 Participants identified a system of service provision, which was fraught with corruption. The discussion further evolved into examples of how the lack of accountability emboldens individuals to either deny services or game the system for personal or collective gain. Focus group participants identify ways in which at the institutional level government agents, international donors, and the research community engage in practices that could be construed as disadvantageous to PLHIV. Finally, participants identify ways in which institutions could provide greater PLHIV protections and ownership of the response system.

**Table 1:** Emergent thematic levels

Thematic Levels		
	Service providers	Institutional
What is experienced	– Theft of resources designated for PLHIV – Misdirection of resources from PLHIV to the public – Denying services due to ethnicity and HIV	– Bribing officials to amend/issue legal documents – Misdirection of resources from PLHIV to the public – Difficult to access service system
How it manifests and affects PLHIV	– Heightens the cost burden of the diagnosis – Longer hospital wait times – Hard to access bank loans	– Lack of support services – Consumers are denied ownership of the response system
Proposed solutions	– Representation within the response system – Equity	– Legal protections of PLHIV Representation within the response system – Transparency

### Service providers

This section presents narratives discussing experiences with service providers, including health care workers, community workers, and bankers. Given the differences in the roles and functions of facility-based health workers and community-based health workers, data on these two groups are presented separately. In addition, given the Kenyan Government’s integration of economic empowerment interventions as a component of the HIV response system [3] and the critical role income and assets play in the health and well-being of impacted households, participant discussions that revolve around banking representatives are included in this section.

In discussions revolving around facility-based healthcare providers, participants described situations where workers denied them services. Specifically, female respondents from Group D discussed how the intersection of HIV, stigma, and tribalism in Kiambu County resulted in a delay of health services. In addition to stigmatization by doctors and other hospital workers, these women described an instance where hospital staff were heard to attribute this delay of service to their tribal affiliation. Here, the women speak of arriving at the hospital early only to have other patients seen before them…

*Respondent 1: That one was attended to, then the third one was called, and they kept calling others in for treatment, until they got to the tenth person, and I was just sitting there. I started to ask why I am not being called in yet I was there first. I found my file on the table but it had been kept aside. When I asked, they told me that I was bringing a disturbance, step outside come in only after I have called you in.*

*Respondent 2: I was there that day and she is not the only one who was affected, I too was affected.*

*Respondent 1: So I went outside and I overheard the social worker and the doctor, I don’t know who else was in the room but it’s just the people who work there, I overheard them say, “That one is a Luo (tribe), she will have to wait”. I was very hurt. I went in and asked them, “doesn’t a Luo deserve treatment?”*

It should be noted that upon further probing, respondents from this group did confirm that this denial of service was due to discrimination based on both their HIV status and tribal affiliations: “*here in Thika District, sometimes people are discriminated against because of their tribe”.* Group D. In instances such as these, hospital staff prioritized care for patients with a similar tribal affiliation by delaying treatment to patients from different ethnic groups. However, this group also acknowledged that the system is improving and the situation is not as dire as it once was*.*

Further, participants noted service delivery differences across different service delivery settings. In particular, Group D provided examples to illustrate county variations in public HIV care. It should be noted that theoretically county level services should follow comparable administrative, payment, and care procedures. Participants noted how stark differences in costs and services are unfair, and arouse their suspicions about the proper use and disbursement of national resources that hospitals receive to serve them. These regional differences are especially pertinent given well documented instances of *‘tribalism’* and regional disparities in national resource allocations [19, 20] previously noted:

*Respondent: I am part of a support group in Maragwa. When you go to the clinic in Maragwa, the first thing you are given tea because you arrived early, it is assumed that you have had nothing to eat. If lunch time finds you there you are given lunch. When it’s time to go home you are given two hundred shillings for transport… If you go to the hospital there suffering from the flu, you won’t have to buy medicine. You do not even need to buy a card. However, here where we live, we do not see things like those. They do not reach us. We wonder where they go to.*

In addition to regional service delivery differences, variations in the way providers treat PLHIV were noted between public and private care facilities “*So I went to the private health center called Mulumba (St. Mulumba) and I am normally treated very well. I was very discouraged at the government run ones”* Group D. Although the circumstances surrounding the quality of treatment provided at the public center are unclear, when consumers feel unwelcome, they may not seek needed treatment thereby compromising adherence and retention into care. Participants continued to describe their skepticism about the proper use of resources when they are charged for services that should otherwise be covered by government benefits and therefore offered free of charge to them:

*Respondent: Here even to get a card (medical card) you need money.*

*Respondent: Even if you go there (Thika Hospital) critically ill, you must buy a card before you are attended to.*

*Respondent: And that’s in a government hospital.*

In addition to these auxiliary charges participants were also curious as to why PLHIV are often l turned away by providers when they are ill, “*another thing in this hospital is, when you go on a Saturday or a Sunday and they see your card has CCC*^1^*written on it, they tell you to come back on Monday.* Group D. Thus, participants illustrated how the quality, accessibility, and costs of care vary widely despite government efforts to fund and standardize services and also noted variations in the way providers treated PLHIV. Their conversations therefore centered around reasons why PLHIV did not receive comparable treatment across all public centers, and where the resources clearly intended for them were directed.

Community health workers (CHW) are a critical part of the HIV support system in Kenya. Respondents discuss how community workers benefit from concrete supports that are meant for PLHIV, which leads to greater out-of-pockets costs. One participant describes a situation where a CHW stole blankets intended for her children:

*Respondent: I want to ask something, these CHW, for example there was a time they asked us to fill forms where I live. The lady asked for a hundred shillings for each child. I gave 300 shillings for my children and returned the form to her. Only one child was issued a blanket, the other two got none, and remember we paid a hundred shillings for each form. As the CHW she said she had people she was caring for and took 3 blankets to take to them. Later she was asked whether she had taken those blankets to the people and it turned out she hadn’t. The blankets were taken from her house by the Headman. She said they would have to wait for her to bring them because she was already using them in her house. You see, they hide these things from the people they are expected to benefit.*

*Respondent: And that person gets paid to work, they are salaried.*

*Respondent: They ask us for all this money and they forget we have to work very hard to get it, even go without some things so that they can forward those forms for you*. Group B

Participants expressed outrage and disappointment that a paid professional would exploit a patient, especially one who is also experiencing financial struggles. Conversation continued and participants speculated that CHWs might engage in these behaviors because many are not HIV infected and do not relate to the patients they serve. Recruiting and training CHWs from the PLHIV community was proposed as a counter to this exploitation. Participants argued that CHWs who share a diagnosis with their clients might be more inclined to empathize with and therefore provide better services to the community as described by one participant in Group B: “*If CHW has HIV it’s okay … they will relate because the person they are helping has HIV too.*” Group B.

Economic empowerment is a strategy that has been used in the public and private sectors to address the HIV epidemic. Given the economic burden of a sero-positive diagnosis and the high poverty rates in the region, income-generating activities were discussed by all groups as interventions that strengthen this population both economically and socially. For instance, participants from Group A discussed the merits of their rabbit rearing business which served a threefold purpose: a much needed and affordable protein for group members, a source of income when excess products are sold to local communities, and finally as an activity that keeps the group socially engaged and connected.

Given the preeminence of income in the fight against HIV, financial institutions play a key role in the response system. Participants from groups B, C, and D discussed how proposals written by PLHIV meant to bolster their economic functioning are often un-funded or when funded the resources are directed towards non-affected groups. Participants argue that a sero-positive status, their low-income status, and tribal affiliation is used to discriminate against funding for group *projects, “as members we write proposals. But when you write this proposal, you hear that another group benefited but the one that has HIV people questions are asked about how they will repay a loan”* Group B. Respondents are indeed committed to securing their economic well-being and do make the necessary efforts to attend the relevant trainings in the hope of receiving financial supports which are not always forthcoming, “w*e were trained for 6 months and we did not get any support”* Group C*.*

Participants proposed several solutions to the issues they had identified. To reduce discrimination, group members discussed the importance of having a representative voice and equity in the service provision system. Representation is viewed as a way in which agencies can be held accountable during service provision. Specifically, respondents highlight the benefits of including their peers as representatives on these government councils.

*Respondent: I would also like us to talk about the government, yes the government is saying that its giving us support but those who have been given the mandate to help us are benefiting themselves.*

*Respondent: For example the government gives food to those who are HIV positive and those who are supposed to supply the food are selling the food, those who are in charge are not affected like us and the people who are chosen are not HIV positive… In case there is any support that is given we should have representatives…we should know the representatives very well.* Group C

The solution of representation was further elaborated when the group members continued their discussion:

***Interviewer: There should be a representative?***

*Respondent: Yes, the money should not be distributed by the government.*

*Respondent: One of us should be involved in the distribution of that money.*

*Respondent: We should be the ones who are supposed to be given the money.*

*Respondent: …we also get people who usually come and tell us to fill forms, give our passport photos, ID and those documents are going to be dumped so we don’t believe anyone.*

*Respondent: They make money off of us.* Group C

Earlier, respondents expressed their belief that providers divert resources away from PLHIV because they do not understand or relate to PLHIV. Thus, respondents assume that by having PLHIV involved in decision making and service provision, providers will be less likely to take or misallocate resources, thereby increasing accountability within the system.

### Institutional level

At the institutional level participants perceived a system that is permissive of corrupt government officials, misdirection of national resources intended for PLHIV, and a difficult to access response system. One of the ways this manifests is the collusion of government officials with relatives and non-relatives alike to deny PLHIV of critical economic resources. Again, given the strong association between HIV and economic disenfranchisement, the theft and misappropriation of their meager resources is especially detrimental to their well-being. Participants shared a variety of situations where family members and village elders deliberately colluded with government officials to misdirect resources intended for them. Both genders identified women as being especially vulnerable. For example, one participant from Group B describes how relatives bribe legal officials to deny widows access to land upon the death of a husband by altering official documentation: *“It comes in because as I have told you, the brother has money, right? You the widow are left without any money. Because of corruption, the said brother will go and give whatever amount of money and after a few minutes, the documents will be given to him with his name written on them”.* A participant from the same group added, *“Let me contribute a little, for instance, everywhere in this country there is an assistant chief. The assistant chief knows everyone in their area. They know whose wife you are, they know who has died. If the assistant chief is bribed, your case is closed. It starts with the administration, because the administration in the village is the chief and assistant chief. Whether you go to the chief or to the police station, they all work in the same way, together”.* Group B

A male participant from group A described an experience he had where he helped a female acquaintance secure legal documents: “*There is a lady I went to assist to get a death certificate at the City Mortuary. She had tried to get one for a whole year and every time she would go they would tell her that they already issued one. When I went there…I found out that they had written two death certificates. I asked why they had issued two death certificates, had this person died twice? How can you note two death certificates for one person?”* In this instance the document in question was a death certificate, a document which is critical to the succession process and is used to secure household assets after the death of a family member.

Institutional players were further identified as misdirecting national resources intended for PLHIV. For instance, participants from Group D noted in their discussions instances where food and money donations intended for PLHIV were misdirected away from these intended target groups: “*There was a time I heard that there are donors who send food to Kenya for people who are living the virus. Who does this food benefit because we don’t get it*”. Again, although participants are not a part of the HIV response system, these group members are aware of how resources ought to be allocated and lack of access or receipt of these resources may raise questions about proper allocation and distribution.

How is this corruption at the institutional level experienced by these participants? Participants discussed situations where accountability mechanisms (in place to prevent skimming and false claims) are circumvented and as a result they do not receive potential services and resources. Group C participants describe an instance where they were asked to cooperate with information gathering efforts by a government group seeking external donations. Presumably, the group needed a count of PLHIV to justify their request for funding:

*Interviewer: When you sign somewhere do you usually ask why you are signing?*

*Respondent: Even if we ask we are going to be cheated.*

*Respondent: We are usually told that there is funds that are about to be released and the donors want to know how (many) members we have, write your name and sign, since you want to get support then you will just sign, since the donor is not there to know the only proof that he can get is by me writing my name and signing and that’s why we have said, it’s good that you have a recorder so that the donors can listen to us and not you, the donors should not give the funding to those who are not affected because they do not give us the funds.* Group C

Although participants seemed aware that their cooperation might not yield any direct benefit to them (and in fact, might only serve others’ personal gains), they complied with the request anyway. Given their tremendous financial needs, many felt as though they could not risk not cooperating. Thus, although PLHIV are aware of the potential use of their exploitation for others’ personal gain, they might also often be unwillingly complicit in corruptive behaviors because of their own dire economic statuses. This engenders a feeling of hopelessness, when PLHIV know that the promised assistance may never materialize.

Feelings of mistrust as a result of exploitation were not only reserved for government officials. Participants also detailed their growing mistrust with the research community. They perceive an imbalance in this relationship with the scales tipped towards the researchers’ favor. To protect their own interests, PLHIV have begun demanding monetary compensation prior to consenting to research activities, “…*there are a lot of people who call on us…they then tell us it is possible to be helped. Then you live with that hope…then you live waiting and that is the reason why these days when you ask to interview a person living with HIV they ask you for money. Because people have realized that they are being used”* Group D*.* Another group member offered a poignant statement on the disconnect between the data collected by researchers and the resulting interventions that do not seem to have a direct impact on her life, “n*ow, when you interview groups and go back abroad, we the ones you have interviewed are not the ones who benefit from the research. Doesn’t that hurt?”*

To address some of these situations participants first identify individual wealth protection mechanisms that they can undertake to protect their economic resources and to circumvent the collusion of family members with corrupt government officials. Participants from all four groups discussed at great lengths some of the strategies they should apply to protect themselves from economic exploitation. These include using the legal system, the use of both oral or written wills, and divulging the location and quantity of household wealth holding to children early on to stave off disinheritance after their deaths.

Participants also proposed several solutions to address the institutional loop holes identified in the system including consumer accountability and having a representative voice at the table. Such an approach would not only deal with the misdirection of services but also promote the availability of services that are targeted and tailored to this population’s specific needs. In the quote below a participant in Group B posed a question to the group pointing towards the need for accountability in funding streams. “*May I ask a question? Is it possible for example when people are writing proposals, there are those who sit down and decide that HIV people need this and this, is it possible two have at least two HIV Positive people in those meetings?”* This sentiment is echoed by participants in part of a conversation that was initiated by the interviewer. This discussion points to a level of accountability that PLHIV would inject into the system “*if this money was in the custody of people living with AIDS in Kenya, it would get to those who are affected because they know all the difficulties of this disease”* Group A.

Participants reported feeling infantilized by the system and want ownership of the research process to ensure that the results address critical problems that they encounter. To do so, respondents noted the importance of community participatory research approaches:

*Respondent: when you are doing your research please tell them not to start at the top. When you start at the top it doesn’t reach them (PLHIV), it doesn’t pay*

*Respondent: It doesn’t reach us. Come to the grass root. Like now you have come to the grass root. If you started at the top you couldn’t know anything*

*Respondent: Even those projects they (donors) think they can start, they should start from the ground, send their people here, they start from the ground.* Group A

Thus by starting “at the top” participants feel as though research studies or other externally initiated projects might not be addressing the critical problems that they encounter and, thus, the results are not likely to benefit them.

## Discussion and implications

These findings emerged from a larger study designed to understand intergenerational wealth transmission in families impacted by HIV in Kenya. Perceived corruption emerged as a salient theme during participants’ discussion of their experiences. Given the research team’s responsibility to report findings as entrusted to them by study participants, this particular sub-study further explored consumers’ experiences and perceptions of corruption. Using transparent and rigorous data analysis procedures, perceived instances of corruption were identified among direct care providers in health care and other service systems and at the institutional level among both local and high-ranking public officials. Corruption is widespread and, thus, a high priority concern among people living in sub-Saharan Africa [39]. In this study, participants described the corruption throughout Kenya’s HIV response system in their own words. Corruption manifests as a variety of behaviors among public decision makers, but differs depending on the setting. Within healthcare organizations, other entities that serve PLHIV, and within the larger governmental and international institutions, participants described how they believed that providers diverted medications, food, and other resources intended for PLHIV, charged for services that were perceived to be free, delayed treatment, or outright denied benefits to PLHIV. Many of these actions could have been motivated by public officials’ or health care workers’ personal economic interests; however, in some of the cases described, these officials or workers did not directly benefit. Rather, other patients or citizens from similar tribal groups, or those who are not directly affected by HIV benefitted from their actions by accessing resources or services that may not have been merited. Although corruption is typically motivated by direct personal gains, our study illustrates how corruptive behaviors might also be motivated by the potential to extend such gains to one’s own social group. Based on participant perceptions, in many cases, decision makers might be justifying their actions based on stereotypes about PLHIV or tribal divisions.

### Mitigating corruption – implications for intervention

Study findings highlight the potential for several policy or programmatic interventions for mitigating corruption within Kenya’s HIV response system. Chiefly, participants advocated for greater ownership of the HIV response system in the form of PLHIV representation. Members of all four peer support groups involved in this study believe that including PLHIV in decision-making could hold institutions accountable thereby creating a more effective service delivery system. Citizen participation (and specifically participation of other PLHIV) also encourages greater transparency in the way that resources are allocated and services created. Addressing information asymmetries would not only address the current system inaccessibility but would also empower the community. In other countries, transparency measures and opportunities for citizens to have a voice in administrative and policy decisions have empowered citizens to challenge corruption and ultimately improved satisfaction with the health care system [21, 22]. These present findings therefore add to the growing literature that identifies advocacy among individuals and families impacted by HIV and AIDS to be a useful tool in drawing attention to harmful practices in the HIV response infrastructure; consistent with this movement, communities in Kenya demand greater control over programmatic interventions both at the national and local levels.

In addition to greater accountability and transparency within Kenya’s public and private institutions, participants also identified the need for additional support for controlling their assets and economic security. PLHIV are especially vulnerable to asset loss when they are set to inherit wealth from a family member. Additional legal protections that safeguard PLHIV from property grabbing could ensure participants rightfully inherit assets. In addition, further assistance with inheritance planning could help PLHIV ensure their assets are appropriately directed in accordance with their wishes upon their death. Together, legal protections and financial planning assistance could improve individuals’ control over their assets.

Our findings also highlight ethical and accountability implications for the international community. Participants’ concerns that international aid does not reach them point to the need for international donors to be aware of potential resource diversions and the negative consequences for PLHIV. For instance, USAID developed tools for assessing corruption in country and tailoring anti-corruption strategies [40]. Tools such as these that recognize each society’s unique socio-cultural, political, and economic environment hold promise in addressing this issue. In collaboration with government officials, more proactive fiscal and programmatic monitoring and other accountability structures could help ensure international aid reaches the intended targets. In addition, international researchers studying HIV treatment, service needs, and experiences of PLHIV should consider their local contributions. Consumers have a long history of mistrust with researchers – researchers who win external funding to study the HIV pandemic arrive in-country and gather data directly from PLHIV. Yet, participants rarely see the results. Furthermore, programs and policies are designed based on the interpretation of that data by people outside of the community [26]. Producing ethical and responsible research that fills a local need calls for well-designed scientific participatory approaches.

Finally, anti-corruption measures are unlikely to be successful in Kenya without continued efforts to reduce stigmatization and discrimination based on HIV status and ethnic membership. Given that disclosure of HIV sero-status is integral to procuring resources from service providers (41) pervasive stigmas and stereotypes create a social context that allows decision makers to rationalize corruption [6]. So until misinformation surrounding a HIV diagnosis is eradicated and the individual worth of persons affected by the virus is socially elevated, corrupt acts targeting this population may continue. At the institutional level, appealing to public service motivations and promoting strong ethical values among decision makers [17] will hopefully begin to address this situation.

### Limitations

Because this study was not designed with the specific intent of exploring corruption, we acknowledge that we may have missed other facets of this phenomenon. Although participants shared their personal experiences with corruption as service consumers, they might not be privy to observing (or confirming) all forms of corruption, especially behaviors that occur among higher-level administrators and officials. Thus, we recognize that these data do not provide objective evidence of corruption but do provide preliminary evidence of the influence of consumers’ perceptions about corruption on resource acquisition, health service access, and outcomes. In addition, the study design precludes any local or national generalizability on the extent to which corruption occurs or affects PLHIV. However, these perspectives gathered from PLHIV who are well positioned to describe their experiences with the system are vital and should be considered in our scrutiny of the HIV response system. Although corruption is perceived to be widespread throughout Kenya, follow up research is needed to determine the magnitude of the problem, how frequently it occurs, and the resulting impact on PLHIV. In addition, future research is needed that tests the effectiveness of anti-corruption measures targeting government agencies as well as providers in the HIV response system.

## Conclusions

Participants place great importance on the theme of corruption as it relates to their overall well-being. This study expands on prior literature documenting the negative consequences of corruption on national economic development and health by illustrating the impact on the daily lives and well-being of PLHIV. Specifically, our findings highlight how corruption in Kenya’s health system and governmental institutions may be driven, in part, by HIV and tribal stigmas. Based on participants’ accounts, corruption throughout the system results in fewer resources for PLHIV. Corruption in the form of resource misappropriation or theft is especially deleterious given the high cost burden already borne by the study population.

## Abbreviations

AIDS: Acquired immune deficiency disorder
ART: Anti-retroviral therapy
CACC: Constituency AIDS Control Committee
CBO: Community based organization
CCC: Comprehensive Care Centers
CHW: Community health workers
HIV: Human immunodeficiency virus
HTC: HIV testing and counseling
KES: Kenyan shilling
OVC: Orphaned and vulnerable children
PLHIV: People living positive (with HIV)
PMTCT: Prevention of mother-to-child transmission
STI: Sexually transmitted infection
USAID: United States Agency for International Development

## Footnotes

1 Comprehensive Care Centers (CCC) are medical centers charged with the delivery of HIV medical services including counseling, nutrition, and other support services.
